# *Streptococcus lutetiensis* neonatal meningitis with empyema

**DOI:** 10.1099/acmi.0.000264

**Published:** 2021-09-13

**Authors:** Allen T. Yu, Kate Shapiro, Christy A. Beneri, Lisa S. Wilks-Gallo

**Affiliations:** ^1^​ Medical Scientist Training Program, Renaissance School of Medicine at Stony Brook University, Stony Brook, NY, USA; ^2^​ Department of Pediatrics, Renaissance School of Medicine at Stony Brook University, Stony Brook, NY, USA

**Keywords:** empyema, MALDI-TOF, *Neonatal sepsis*, neonatal meningitis, *Streptococcus lutetiensis*

## Abstract

*
Streptococcus lutetiensis* has been known to cause sepsis in adults, but only one case regarding neonatal sepsis has been reported internationally, with no sequelae. We report the first case of neonatal bacteremia and meningitis with empyema caused by *S. lutetiensis* in the United States.

## Case report

A 28-day-old baby boy presented with fever, irritability, and inconsolability prompting the parents to seek medical attention. He had no other symptoms, including no upper respiratory symptoms, cough, vomiting, diarrhoea, or rash. He had been home and well since discharge from the newborn nursery. There were no identified sick contacts; however, the mother reported multiple visitors recently, the family has young pet chickens at home, and the patient’s stepsister visited a friend’s house with baby cows. He was born at term, weighing 3300 g. Maternal prenatal culture was negative for group B streptococcus. Foetal anomalies had been noted on early ultrasound including a two vessel cord, choroid plexus cyst, and short humerus and femur lengths. The choroid plexus cyst and short humerus and femur lengths resolved in later ultrasounds. Labour was uneventful, with spontaneous rupture of membranes for 6 h and thick meconium. At birth, the baby had 1 and 5 min Apgar scores of 9 and 9. Patient was discharged on post-partum day one without complications.

On presentation to the emergency room, vital signs were a temperature of 100.9 ° F, heart rate of 187 min^−1^, respiratory rate of 46 min^−1^, blood pressure of 101/58, and O_2_ saturations at 100 % on room air. On physical exam, he was well-appearing and without focal findings. A full sepsis workup was obtained. Therapy with intravenous ampicillin (75 mg kg^−1^ every 6 hours) and ceftazidime at meningitic doses (50 mg every 8 hours) was started. Laboratory studies revealed white blood cells at 10 360 µl^−1^ (neutrophils, 52 %, lymphocytes 30 %, monocytes 14 %, eosinophils 3 %, immature granulocytes 0.2 %). Urinalysis was negative for nitrates and leucocyte esterase. Influenza, respiratory syncytial virus, and COVID-19 testing were negative. Blood cultures and urine cultures were sent. Cerebrospinal fluid (CSF) was obtained by lumbar puncture for culture, but there was inadequate sample size for additional CSF analysis.

Initial blood and CSF Gram-stain showed Gram-positive cocci in pairs and chains. BioFire Filmarray PCR reported *Streptococci* sp., not A, B, or *pneumoniae*. Both CSF and blood were cultured on chocolate agar and 5 % sheep blood agar. Subsequent colonies were analysed via MALDI-TOF, which identified the bacteria as *Streptococcus lutetiensis *, when compared to the negative controls of formic acid and matrix, as well as the positive controls of *S. aureus, E. coli, and C. krusei*. Further testing showed that it was sensitive to ceftriaxone (0.032 µg ml^−1^), penicillin (0.047 µg ml^−1^), and vancomycin. All culture and breakpoint protocols were performed according to CLSI guidelines. Ceftazidime was replaced with intravenous ceftriaxone (100 mg kg^−1^ every 1 d). Urine culture was negative. With the pathogen identified and susceptibilities known, the infant was continued on ceftriaxone alone. The fevers persisted, so brain MRI with and without contrast was performed on hospital day 5, revealing a small subdural empyema. The baby was transferred to the Paediatric ICU for close monitoring and neurological checks. Neurosurgery was consulted but surgical drainage was not pursued based on size of the empyema and patient’s reassuring clinical status. C-reactive protein and procalcitonin were 8.3 mg dl^−1^ and 2.69 ng ml^−1^, respectively. The fever resolved on day 6 of treatment. Follow up blood cultures from day 2, day 3, and day 6 of admission were all negative. Cardiac echocardiogram to rule out endocarditis was negative. Repeat MRI showed resolution of the empyema on hospital day 9, and the patient was discharged on hospital day 11 on ceftriaxone via Broviac line. The patient completed a 4 week course of ceftriaxone, and the Broviac was removed.

Since discharge, the infant experienced two seizure-like episodes, but with no associated fever, cyanosis, hypotonia, vomiting, diarrhoea, or change in feedings. Vital signs and labs including complete metabolic panel and blood count on both episodes were within normal limits. EKG and video EEGs were normal, and repeat MRIs of the brain with and without contrast were unremarkable for concerns of source of seizure or for infection. He is doing well without antiepileptic medications and follows with neurology and neurosurgery.

## Discussion

Neonatal sepsis is a significant cause of morbidity and mortality. Of the 0.98 cases of neonatal sepsis per 1000 live births, 43 % are due to group B streptococci and 29 % due to *E. coli* [1]. The organism isolated in our patient is classified as a group D streptococci and is a subtype of *S. bovis *. While group D streptococci, such as enterococci, are recognized as causes of neonatal sepsis, *Streptococcus bovis* is considered a rare cause of infection [2]. The exact prevalence of infection may be underestimated due to the misidentification of many clinical group D streptococci isolates as enterococci, which was formerly classified as part of the group D, or viridians, streptococci [3].

The taxonomic status of *S. bovis* has been undergoing active refinement due to the advancement of molecular sequencing and genetic techniques, and recently *S. bovis* may possibly be equivalent to *S. equinus *, and became grouped as the *Streptococcus bovis/Streptococcus equinus* complex (SBSEC) [4]. For simplicity, we refer to SBSEC as *S. bovis* for the remainder of the text. Older literature demonstrates that *S. bovis* biotype I has the ability to hydrolyse starch and ferment mannitol, while *S. bovis* biotype II does not [5]. Biotype II is further divided into sub-biotypes II/1 and II/2, based on phenotypic testing for production of various sugars and amino acids and fermentation abilities [6]. Genetic analysis has now sub-divided *S. bovis* into five different species (* S. equinus *, *S. gallolyticus *, *S. infantarius *, *S. pasteurianus *, and *S. lutetiensis *) [7]. Further dissection of the *S. bovis* group by analysis of manganese-dependent superoxide dismutase gene resulted in the reclassification of *S. infantarius* subsp. *coli* to *S. lutetiensis* (* S. bovis* biotype II/1), and *S. bovis* biotype II/2 as *S. pasteurianus* [8]. The isolates from the current case are presented with the current taxonomic classification as *S. lutetiensis *.

Identification of the correct isolate for each subspecies is crucial for choosing the appropriate antibiotic therapy, as well as management of potential sequelae. *S. bovis* biotype II, the organism identified in our infant, is associated with adult bacteremia, but can also cause invasive infection in neonates and infants [5, 9]. Beginning with the first recorded case in 1978 by Headings *et al*., there have been 55 reported cases of neonatal sepsis caused by *S. bovis* biotype II [10–14]. While the nine cases of neonatal sepsis caused by *S. bovis* did not biotype the clinical isolates prior to the year 2000, every subsequent case, except for one, identified the organism as *S. bovis* biotype II/2, or *S. pasteurianus *. In addition, it has been reported that *S. pasteurianus* neonatal sepsis can cause delayed central nervous system complications, as well as endocarditis and pneumonitis [10, 11].

While *S. bovis* is part of the normal colonic and oral flora, the source and mechanism of neonatal *S. bovis* infection remains elusive [15]. *S. bovis* has been isolated previously from normal vaginal secretions as well as from the rectum [16, 17]. However, our patient qualifies as late onset neonatal sepsis, and environmental exposure should be considered, including the potential contact with livestock. We suspect this is post-natal acquisition of the pathogen. While our patient did not have any sick contacts, there were numerous potential routes of infection: the patient had multiple visitors recently, the family raises young pet chickens at home, and the patient’s stepsister visited a friend’s house with baby cows. As noted in a prior case series, it is also possible that disruption of the gastrointestinal mucosa by gastroenteritis facilitates bacterial translocation, and may explain the pathogenesis though our patient did not have such gastrointenstinal symptoms at presentation [18]. In addition, *S. bovis* colonization of the ear and throat has been reported and human to human transmission is not impossible [19].

As of this writing, only one international case of neonatal sepsis known to be secondary to *S. bovis* subtype II/1, or *S. lutetiensis *, has been reported in the literature [20]. The previously reported case was also a 28 day old infant who presented with fever and elevated inflammatory markers. After antibiotic therapy, that previous infant defervesced on hospital day 2, and the patient was discharged to complete a 14 day course of ampicillin/sulbactam without sequalae. To our knowledge, our patient is the second reported case of *S. lutetiensis* neonatal sepsis and the first associated with meningitis and subdural empyema. Thus, it is clinically important to accurately identify the biotype of the bacteria using a rapid diagnostic method, such as MALDI-TOF, in order to monitor for possible adverse sequelae, and care must be taken when *S. lutetiensis* is isolated as the cause for neonatal sepsis. Our case suggests that *S. lutetiensis* may be associated with subdural empyemas, in addition to causing meningitis. As in our case, quick identification of the pathogen through rapid diagnostic methods can support decisions for targeted anti-microbial therapy which will treat the infection more effectively and lead to improved patient outcomes.
